# Choice of High-Dose Intravenous Iron Preparation Determines Hypophosphatemia Risk

**DOI:** 10.1371/journal.pone.0167146

**Published:** 2016-12-01

**Authors:** Benedikt Schaefer, Philipp Würtinger, Armin Finkenstedt, Vickie Braithwaite, André Viveiros, Maria Effenberger, Irene Sulzbacher, Alexander Moschen, Andrea Griesmacher, Herbert Tilg, Wolfgang Vogel, Heinz Zoller

**Affiliations:** 1 Medical University of Innsbruck, Department of Medicine II, Gastroenterology and Hepatology, Anichstr. Innsbruck, Austria; 2 Central Institute for Medical and Chemical Laboratory Diagnosis, Innsbruck University Hospital, Anichstr. Innsbruck, Austria; 3 Medical Research Council (MRC) Human Nutrition Research, Elsie Widdowson Laboratories, Fulbourn Road, Cambridge, United Kingdom; 4 Medical University of Innsbruck, Department of Medicine I, Gastroenterology, Endocrinology and Metabolism, Anichstr. Innsbruck, Austria; 5 Medical University of Vienna, Department of Pathology, Währingergürtel, Vienna, Austria; Lady Davis Institute for Medical Research, CANADA

## Abstract

**Background:**

Ferric carboxymaltose (FCM) and iron isomaltoside 1000 (IIM) are increasingly used because they allow correction of severe iron deficiency in a single infusion. A transient decrease in serum phosphate concentrations is a frequent side effect of FCM.

**Aim:**

To characterize this adverse event and search for its predictors in a gastroenterology clinic patient cohort.

**Methods:**

Electronic medical records of patients attending the University Hospital of Innsbruck were searched for the keywords ferric carboxymaltose or iron isomaltoside. Eighty-one patients with documented administration of FCM or IIM with plasma phosphate concentrations before and after treatment were included.

**Results:**

The prevalence of hypophosphatemia (<0.8 mmol/L) increased from 11% to 32.1% after treatment with i.v. iron. The hypophosphatemia risk was greater after FCM (45.5%) compared with IIM (4%). Severe hypophosphatemia (<0.6 mmol/L) occurred exclusively after FCM (32.7%). The odds for hypophosphatemia after i.v. iron treatment were independently determined by baseline phosphate and the choice of i.v. iron preparation (FCM vs. IIM—OR = 20.8; 95% CI, 2.6–166; p = 0.004). The median time with hypophosphatemia was 41 days, but prolonged hypophosphatemia of ≥ 2 months was documented in 13 of 17 patients in whom follow-up was available. A significant increase in the phosphaturic hormone intact FGF-23 in hypophosphatemic patients shows that this adverse event is caused by FCM-induced hormone dysregulation.

**Conclusion:**

Treatment with FCM is associated with a high risk of developing severe and prolonged hypophosphatemia and should therefore be monitored. Hypophosphatemia risk appears to be substantially lower with IIM.

## Introduction

Ferric carboxymaltose and iron isomaltoside are iron-carbohydrate complexes, which have shown high efficacy and a low risk of allergic reactions in clinical trials. Even in severely iron deficient patients total iron deficit can be readily corrected in 1–2 infusions. Therefore, the introduction of these iron preparations has prompted the development of new treatment guidelines for patients with iron deficiency anemia due to inflammatory bowel disease, cancer-related anemia and in pregnant women[1–4].

Transient hypoposphatemia is the most common adverse event after treatment of iron deficiency anemia with ferric carboxymaltose (FCM) in patients with inflammatory bowel disease (IBD)[5, 6]. When trials including patients with chronic kidney diseases are excluded, the reported incidence of hypophosphatemia after FCM treatment is 41–70%, as compared to 5–8% after IIM treatment[7–10]. No head to head comparison between FCM and IIM have been reported and it is unknown if these differences are attributable to different pharmacological properties or reflect heterogeneity in study populations or dosing.

Studies further show that after single dosing the effect of FCM and IIM on plasma phosphate concentrations is transient and mean phosphate returns to normal within 4–12 weeks. In clinical practice, repeated dosing may be required in selected patients with ongoing blood loss. In such patients, protracted hypophosphatemia causing osteomalacia and muscular weakness has been reported[11–18].

The decrease in plasma phosphate concentration after FCM infusion is mediated by an increase in the biologically active, intact fibroblast-growth factor 23 (iFGF-23). This leads to reduced tubular reabsorption and renal phosphate wasting. Iron deficiency itself causes an increased FGF-23 transcription, which does not normally result in hypophosphatemia because of simultaneously increased cleavage and deactivation of iFGF-23. Although these biochemical events are well-documented, the iFGF-23-inactivating enzyme which is inhibited by FCM is unknown[19, 20].

The aim of this retrospective study was to compare the propensity of FCM and IIM to cause hypophosphatemia in a real life cohort of gastroenterology clinic patients.

## Patients and Methods

### Patient selection

The study protocol was approved by the Medical University of Innsbruck’s ethics committee (Ethikkommission der Medizinischen Universitaet Innsbruck, study number AN2015-0239 354/4.8). In accordance with the approved study protocol and in accord with Austrian law, no specific consent is required for this retrospective analysis. Additional parameters (1,25 (OH)2-Vitamin D, 25-OH-Vitamin D, Parythroid hormone, C-terminal and iFGF23) were determined in archival blood samples, which were collected for research purposes after written informed consent had been obtained from each patient at the time of collection. Patients who received i.v. iron treatment were identified by an electronic medical record search of documents from the Gastroenterology and Hepatology department of the University Hospital of Innsbruck, Austria for the keywords ‘ferinject’, ‘carboxymaltose’, ‘monofer’ and ‘isomaltoside’. During the search period from 01.01.2002 to 01.10.2015, a total of 539 documents were identified. These documents referred to 202 individual patients for ‘ferinject or carboxymaltose’ and 123 patients where ‘monofer or isomaltoside’ was mentioned. Inclusion criteria were documented infusion of either FCM or IIM, and the availability of plasma phosphate concentration for each patient up to 6 months before the infusion date and up to 12 months after the infusion date. These inclusion criteria were met in 81 patients, where 27% (55 of 202 patients) of the FCM group and 21% of the IIM group (26 of 123 patients) could be included in the study. The choice of iron preparation used was based on availability, where IIM became available after September 2012 at our institution. Hence, all 46 study patients treated between February 2010 and August 2012 have received FCM. Of 35 patients treated between September 2012 and June 2015, 9 patients were treated with FCM.

To study the duration of hypophosphatemia, all phosphate measurements were extracted from the electronic medical records and analyzed. From the group of patients who became hypophosphatemic, 9 patients were excluded for the following reasons: (i) only one phosphate measurement after i.v. iron treatment was available (2 patients), (ii) subsequent treatment with another i.v. iron infusion before normalization of phosphate (3 patients) and (iii) patient had hypophosphatemia before i.v. iron administration (4 patients).

Plasma calcium, phosphate, alkaline phosphatase and albumin concentrations, whole blood hemoglobin concentration and serum iron parameters (including serum iron, ferritin, transferrin and transferrin saturation), were determined by routine laboratory tests at the Central Institute for Medical and Chemical Laboratory Diagnosis, Innsbruck University Hospital and parameters were extracted from the medical records. Plasma calcium (inter-assay imprecision CV <1.2%), the phosphate concentration (inter-assay imprecision CV <2.1%), the alkaline phosphatase concentration (inter-assay imprecision CV <3%), serum iron parameters iron, ferritin and transferrin (inter-assay imprecision CV <2%, <5% and <3%) and albumin concentration (inter-assay imprecision CV <4%) were determined on the modular system (Roche Diagnostics GmbH, Mannheim, Germany) and hemoglobin concentration were determined on the Sysmex XE-5000 hematologic analyzer (Sysmex Corp., Kobe, Japan, inter-assay imprecision CV <1%).

The severity of hypophosphatemia was graded according to the Common Terminology Criteria for Adverse Events (CTCAE, version 4.0)[21] In a subgroup of 34 patients a stored serum sample from before and in 29 patients a stored serum sample from after i.v. iron treatment was available for further analysis. Two samples for both before and after were available from 26 patients, which allowed for paired analysis in this subgroup. Parathyroid hormone (PTH) concentration was determined using an electrochemiluminescence immunoassay based on monoclonal antibodies that detects both the N-terminal fragment and the C-terminal fragment of the hormone PTH (Modular, Roche Diagnostics GmbH, Mannheim, Germany; inter-assay imprecision CV <7%). 25-hydroxyvitamin D (25(OH)D) was measured by high pressure liquid chromatography (HPLC) (Chromsystems, Munich, Germany; interassay imprecision CV <6%, standardized with NIST reference material). The assay detects both 25(OH)D2 and 25(OH)D3 and so the total 25(OH)D is reported as the sum of both isoforms. Measurement of 1,25-dihydroxyvitamin D (1,25(OH)_2_D) was performed with the automated IDS-iSYS 1,25-dihydroxy-vitamin D assay (IDS Immuno-Diagnostic Systems, Boldon, UK; interassay imprecision CV <18%). C-terminal fibroblast growth factor (cFGF-23) was quantified by use of an enzyme-linked immunosorbent assay (ELISA) (Biomedica, Biomedica Medizinprodukte, Vienna, Austria; interassay imprecision CV <10%) that detects epitopes within the carboxyl-terminal of FGF-23 with polyclonal antibodies. Intact FGF-23 (iFGF-23) was quantified by an ELISA that detects both the N- and C-terminal fragment (Kainos Laboratories, Tokyo, Japan; interassay imprecision CV <10%).

### Statistical analysis

Data were extracted from the patient records and expressed as means ± standard deviation if normally distributed or for non-normally distributed variables as medians with first and third quartiles. Normality of distribution was determined by Kolmogorov-Smirnov test. Frequencies are reported as absolute numbers or percentages as indicated. Quantitative variables were compared using the paired or unpaired Student’s t-test or the non-parametric Mann–Whitney U or Wilcoxon signed-rank test as appropriate. Contingency tables were tested for significance using the Fisher’s exact or χ^2^-test. A p-value ≤0.05 was considered as statistically significant. All statistical analyses were performed using SPSS Statistics 22 (IBM, Chicago, IL, USA).

## Results

**Fig 1:**
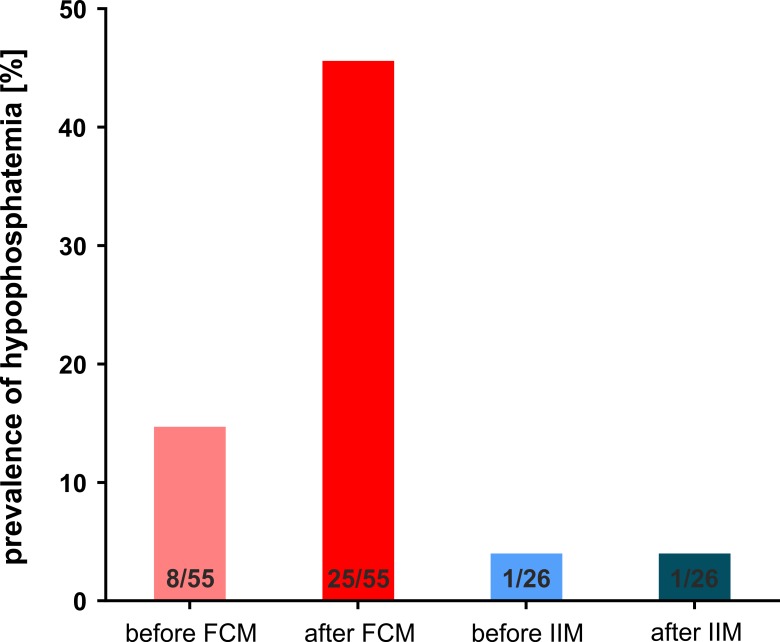
Prevalence of hypophosphatemia before and after FCM and IIM.

**Fig 2:**
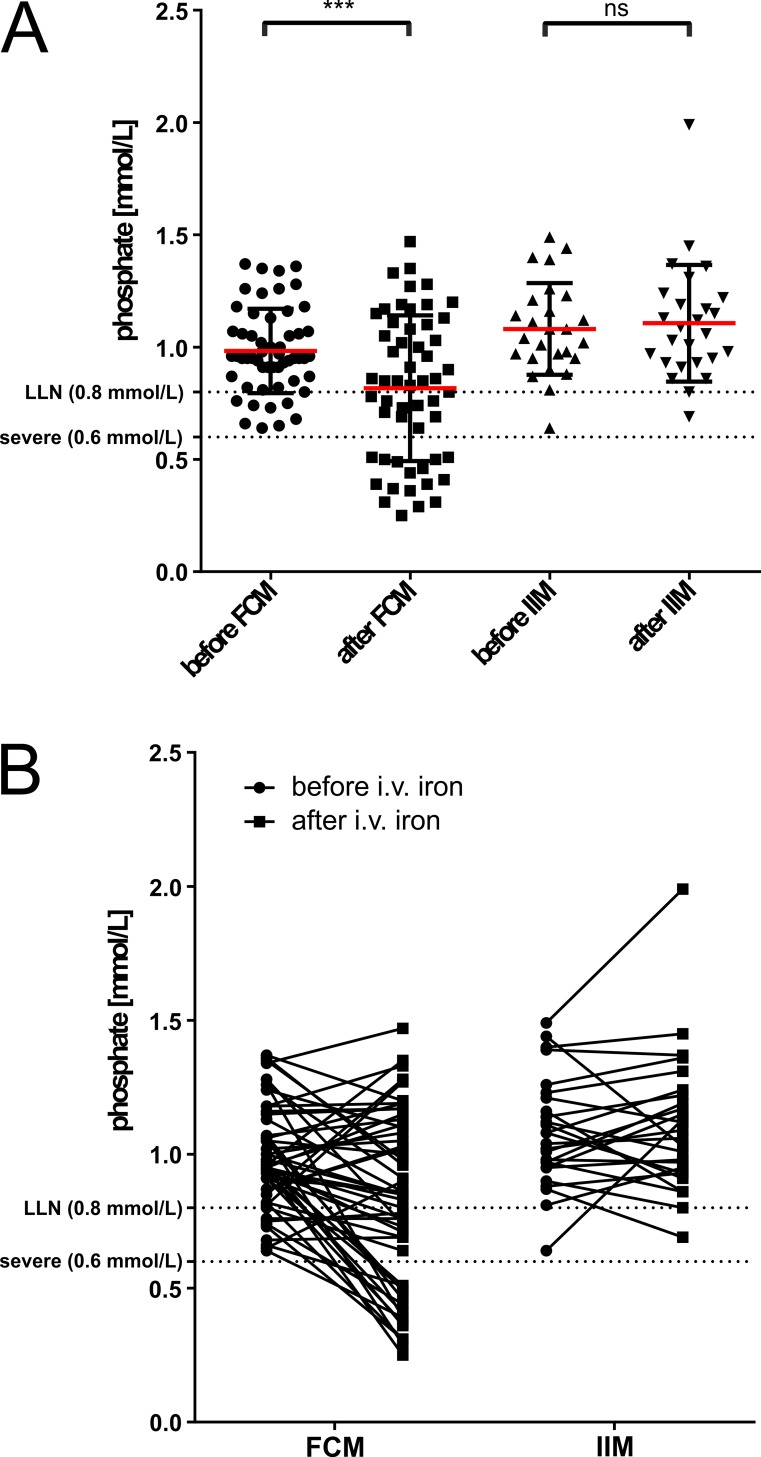
*(A)* Plasma phosphate concentration before and after FCM or IIM. Mean plasma phosphate decreased significantly in the FCM group. Severe hypophosphatemia (< 0.6 mmol/L) was exclusively found after FCM. *(B)* Paired analysis shows a decline in plasma phosphate concentration from before to after the infusion of FCM in the majority of patients. Only one patient developed moderate hypophosphatemia after the infusion of IIM.

The prevalence of hypophosphatemia (<0.8 mmol/L or <2.5 mg/dL) after treatment with high dose intravenous (i.v.) iron in this group of patients was 32.1% (26 of 81). When 9 patients with a priori hypophosphatemia were excluded, the incidence of *de novo* hypophosphatemia after iron therapy was 26.4% (19 of 72). The Common Terminology Criteria for Adverse Events (CTCAE, version 4.0)[21] defines severe hypophosphatemia as 0.3–0.59 mmol/L and life threatening hypophosphatemia as < 0.3 mmol/L. Severe and life-threatening hypophosphatemia occurred exclusively after infusion of FCM with an incidence of 29.1% and 3.6%, respectively (Figs 1 and 2).

Comparing baseline characteristics in patients with and without post-infusion hypophosphatemia after i.v. iron showed significant differences in baseline phosphate, haemoglobin and the choice of i.v. iron preparation (Table 1, Table B in S1 File). This finding was confirmed, when 8 patients with a priori hypophosphatemia were excluded from the analysis. In the hypophosphatemia group more patients received i.v. iron doses >0.5 g (88.4% vs. 65.4%, p = 0.09), but this difference did not reach statistical significance. Regression analysis for baseline predictors of hypophosphatemia revealed that only that the choice of substance (FCM or IIM) and baseline phosphate concentration were independent predictors for the development of hypophosphatemia after i.v. iron treatment (Table 2).

**Table 1:** Clinical and biochemical parameters at baseline in patients who developed hypophosphatemia compared with patients who did not develop hypophosphatemia.

	n	No hypophosphatemia after i.v. iron	n	Hypophosphatemia after i.v. iron	p
Ferric Carboxymaltose	30	54.5%	25	96.2%	< 0.001
Iron Isomaltoside 1000	25	45.5%	1	3.8%	
**Dose:**					0.092
0.5 g	19	34.5%	3	11.5	
1 g	34	61.8%	22	84.6	
>1 g	2	3.6%	1	3.8	
Phosphate (mmol/L)	55	1.07 (± 0.19)	26	0.90 (± 0.16)	< 0.001

**Table 2:** Binary logistic regression analysis for the prediction of hypophosphatemia after treatment with i.v. iron. All variables listed in Table A in the S1 File were tested, but only significant predictors of hypophosphatemia on univariate analysis are listed below.

	univariate		multivariate	
	OR (95% CI)	p	OR (95% CI)	p
age	0.974 (0.948–0.999)	0.045		
Drug	20.8 (2.6–166.7)	0.004	18.7 (2.2–158.6)	0.007
Baseline phosphate [mmol/L]	0.003 (0.0001–0.098)	0.004	0.004 (0–0.164)	0.003

**Fig 3:**
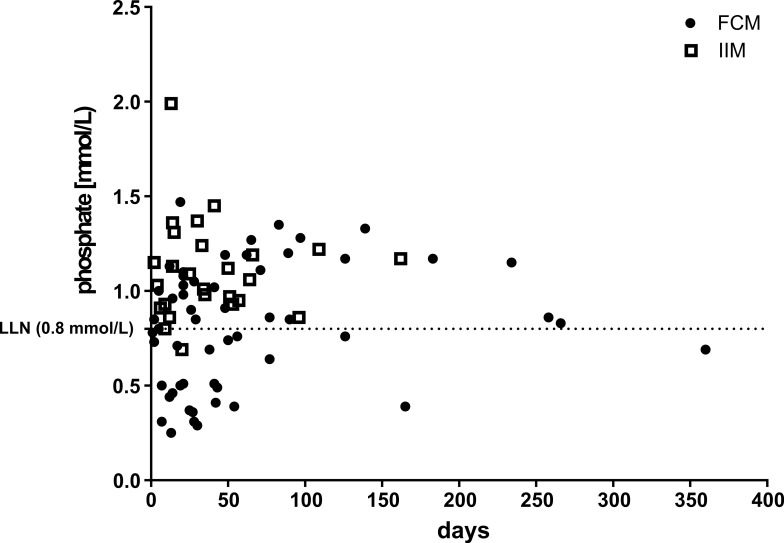
Post-treatment phosphate concentration plotted against the time interval between the plasma phosphate measurement and iron treatment. Different symbols indicate the iron preparation given.

The temporal relation between i.v. iron and hypophosphatemia is shown in Fig 3, which highlights that the lowest phosphate concentrations were found in those patients, who had their first phosphate measurement within the first 60 days after i.v. iron administration. However, mild hypophosphatemia was also found in patients with longer time intervals between treatment and follow-up. In the 17 patients with follow-up phosphate measurements, the median time to normalization of phosphate after a single iron infusion was 84 days (first–third quartile: 62–185 days) and the median time with documented hypophosphatemia was 41 days (first–third quartile: 22–81 days). In all 13 of 17 patients with prolonged hypophosphatemia over 2 months was confirmed by repeated phosphate measurements. Three patients, who received repeated i.v. iron before normalization of plasma phosphate were excluded from this analysis.

To study potential causes of hypophosphatemia in this cohort of patients, the main regulators of phosphate homeostasis iFGF-23, cFGF-23, PTH and Vitamin D were measured in archival samples. The group of patients who developed hypophosphatemia had an increase in median iFGF-23 whereas median iFGF23 decreased in patients who maintained their phosphate within the normal range (34.3 pg/ml vs. -9.7 pg/ml). This difference was statistically significant (p = 0.049, Table 3). In contrast, the changes in serum concentrations of the biologically inactive C-terminal fragment of FGF-23, Vitamin D or parathyroid hormone (PTH) did not discriminate between both patient groups. When markers of phosphate homeostasis were compared in the entire group of patients before and after i.v. iron therapy, median cFGF-23 was significantly lower and median alkaline phosphatase was significantly higher after i.v. iron than at baseline (Table 4).

**Table 3:** Relative changes from baseline to post treatment comparing means or medians in the cohort of hypophosphatemia patients with the group of patients who maintained normal plasma phosphate:

	n	No hypophosphatemia after i.v. iron	n	Hypophosphatemia after i.v. iron	p
Delta serum ferritin (μg/L)	39	80 (9–145)	21	160 (97–286.5)	0.007
Delta phosphate (mmol/L)	55	0.02 (± 0.21)	26	-0.37 (± 0.25)	<0.001
Delta cFGF-23 [pmol/L]	15	-2.6 (-20.8–0)	13	-0.8 (-5 –-0.1)	0.300
Delta iFGF-23 [pg/ml]	19	-9.7 (-19.5–7.3)	8	34.4 (-6.6–76.1)	0.049

**Table 4:** Patients’ characteristics, clinical, biochemical and hematological parameters before and after the infusion of i.v. iron.

	n	baseline	n	follow up (after i.v. iron)	p
n (females)	81	81 (40)			
age	81	49 (38.5–68.5)			
**Underlying diagnosis:**	81				
Crohn’s disease		17			
Ulcerative colitis		19			
Occult blood loss		20			
Other		25			
Hemoglobin [g/L]	81	98.9 (± 22.4)	79	115.7 (± 18.5)	< 0.001
Serum ferritin [μg/L]	79	12 (8–30)	61	138 (54–248)	< 0.001
Phosphate [mmol/L]	81	0.97 (0.91–1.15)	81	0.93 (0.7–1.15)	0.005
cFGF-23 [pmol/L]	34	2.5 (0.8–9.5)	29	1.3 (0.6–3.6)	< 0.001
Alkaline phosphatase [U/L]	78	69 (57–96.3)	78	78 (59–104)	0.004

## Discussion

A transient decrease in phosphate concentrations after administration of FCM and less so IIM was documented in phase 3 trials. Although mean phosphate concentration or incidence of hypophosphatemia was reported, the severity and duration of this treatment-related adverse event for individual patients are unknown[5, 10]. Severe short-term and moderate long-term hypophosphatemia caused by i.v. iron has been associated with muscle weakness, pain and osteomalacia in case reports and small case series[11–18]. The present study confirms that hypophosphatemia has a high prevalence of 32.1% after treatment of iron deficiency with high dose i.v. iron in a real life patient population with gastrointestinal disorders. The single most important risk factor for the development of this complication appears to be the choice of the i.v. iron preparation, where FCM was associated with a 20-fold higher risk than IIM and all 18 cases of severe and life-threatening hypophosphatemia (< 0.6 mmol/L) developed after administration of FCM. The true prevalence of transient hypophosphatemia could be even higher because in some patients the longer time interval between i.v. iron treatment and phosphate measurement could have allowed for spontaneous resolution of low phosphate concentration. The median time to resolution of hypophosphatemia was 84 days. Taken together, this retrospective study shows that after FCM treatment a high proportion of gastroenterology patients experience hypophosphatemia of clinically significant severity and/or duration, whereas only one patient developed mild hypophosphatemia after IIM. These findings should be confirmed in prospective clinical studies with head-to-head comparison of FCM and IIM in patients with different underlying diseases.

Mild transient hypophosphatemia is generally asymptomatic, but even short term severe hypophosphatemia has been associated with clinical complications[22]. These can include bone and joint pain, general weakness, altered mental status and even seizures. Severe hypophosphatemia (<0.6 mmol/L) was present in 32.7% of patients after FCM. Since these symptoms are in part non-specific and can also be present in patients with iron deficiency, clinical symptoms of hypophosphatemia may be falsely attributed to the underlying iron deficiency.

In patients with documented long-term hypophosphatemia triggered by a single or by repetitive FCM infusion, long term complications need to be considered. Bone health is a major concern in patients included in this study. Chronic hypophosphatemia can cause osteomalacia, which is notoriously difficult to diagnose and may be misinterpreted as osteoporosis. Likewise, hypophosphatemia may be attributed to vitamin D deficiency or intestinal malabsorption[23]. Our findings highlight that i.v. iron therapy with FCM is an additional risk factor for hypophosphatemia. The prevalence of bone disease and other long term outcomes were not assessed in this study, but our findings mandate further investigation of bone disease after (repeated) i.v. iron therapy.

One limitation of this study is that urinary phosphate concentrations were not available as in contrast to plasma phosphate concentrations, urinary phosphate was not part of the routine laboratory test panel during the study period. To explore the hypothesis that plasma phosphate concentration is maintained in some patients with renal phosphate wasting at the expense of bone minerals, alkaline phosphatase (ALP) was analysed. The finding that ALP increases after i.v. iron suggests that this treatment can increase bone turnover which may be a response to hypophosphatemia (Table 1). Another potential limitation is the fact that median phosphate concentration was lower in the group of patients who developed hypophosphatemia. This suggests that other risk factors for low plasma phosphate such as malnutrition were more prevalent or more severe among patients who developed hypophosphatemia after i.v. iron. Controlling for such confounders is impossible in a retrospective study and further highlights the need for prospective trials and increased awareness.

A comprehensive study on the mechanism of hypophosphatemia showed that FCM mediates renal phosphate wasting secondary to increased concentrations of iFGF-23[19]. Our findings support this concept because median iFGF-23 concentration increased significantly only in serum samples from patients who developed hypophosphatemia after i.v. iron treatment. The observation that cFGF-23 decreased significantly after i.v. iron treatment suggests that cFGF-23 reflects the transcriptional activity of the FGF-23 promoter, which is induced by iron deficiency via hypoxia inducible factor 1 alpha (HIF-1α)[24, 25]. While both i.v. iron preparations (FCM and IIM) reduce the production of FGF-23 by treating the iron deficit, FCM apparently stabilises the newly synthesised FGF-23 as reflected by the significant increase in iFGF-23[19], only observed in patients who became hypophosphatemic.

In conclusion, this study confirms a high rate of transient hypophosphatemia, and revels that this adverse event is severe and prolonged in a significant proportion of gastroenterology patients after correction of iron deficiency with FCM but less so with IIM. The main implication of our findings is that plasma phosphate concentrations should be checked before and monitored after i.v. iron treatment–especially in patients receiving FCM.

## Supporting Information

S1 File Patients’ characteristics, clinical, biochemical and hematological parameters before and after the infusion of i.v. iron (Table A). Comparison of patients’ baseline characteristics, clinical, biochemical and hematological parameters in the subgroup of patients who developed hypophosphatemia as compared with the group of patients who did not develop hypophosphatemia (Table B). Relative changes from baseline to post treatment comparing means or medians in the cohort of hypophosphatemia patients with the group of patients who maintained normal plasma phosphate (Table C). Comparison of post-treatment biochemical and hematological parameters in the subgroup of patients who were treated with FCM compared with the subgroup of IIM treated patients (Table D). Relative changes from baseline to post treatment comparing means or medians in the cohort of patients treated with FCM or IIM. The low number of IIM treated patients from whom paired stored serum samples were available for iFGF-23 testing does not allow a direct comparison of the effects of IIM and FCM on iFGF-23, cFGF-23, 25 (OH) vitamin D3, 1,25 (OH) vitamin D3 and parathyroid hormone (Table E).
(PDF)

## Abbreviations

25(OH)D: 25-hydroxyvitamin D
BMD: bone mineral density
FCM: ferric carboxymaltose
FGF-23: fibroblast growth factor
GI malignoma: gastrointestinal malignoma
i.v.: intravenous
PTH: parathyroid hormone
IBD: inflammatory bowel diseases
IIM: iron isomaltoside 1000
