# Supplementary material for: Choice of High-Dose Intravenous Iron Preparation Determines Hypophosphatemia Risk
Source: PLoS One. 2016 Dec 1;11(12):e0167146. doi: 10.1371/journal.pone.0167146 (PMC5131956; doi:10.1371/journal.pone.0167146)
Supplement: S1 File — Patients’ characteristics, clinical, biochemical and hematological parameters before and after the infusion of i.v. iron (Table A). Comparison of patients’ baseline characteristics, clinical, biochemical and hematological parameters in the subgroup of patients who developed hypophosphatemia as compared with the group of patients who did not develop hypophosphatemia (Table B). Relative changes from baseline to post treatment comparing means or medians in the cohort of hypophosphatemia patients with the group of patients who maintained normal plasma phosphate (Table C). Comparison of post-treatment biochemical and hematological parameters in the subgroup of patients who were treated with FCM compared with the subgroup of IIM treated patients (Table D). Relative changes from baseline to post treatment comparing means or medians in the cohort of patients treated with FCM or IIM. The low number of IIM treated patients from whom paired stored serum samples were available for iFGF-23 testing does not allow a direct comparison of the effects of IIM and FCM on iFGF-23, cFGF-23, 25 (OH) vitamin D3, 1,25 (OH) vitamin D3 and parathyroid hormone (Table E). (PDF) [file pone.0167146.s001.pdf]

Supporting Information

Table A:

	n	baseline	n	follow up (after i.v. iron)	p
n (females)	81	81 (40)			
age	81	49<br>(38.5 – 68.5)			
**Underlying diagnosis:**<br>Crohn's disease<br>Ulcerative colitis<br>Occult blood loss<br>Other	81	 17<br>19<br>20<br>25			
Hemoglobin [g/L]	81	98.9<br>(± 22.4)	79	115.7<br>(± 18.5)	< 0.001
Serum iron [µmol/L]	79	4.6<br>(3.1 – 8.3)	61	10<br>(5.5 – 15.5)	< 0.001
Serum ferritin [µg/L]	79	12<br>(8 – 30)	61	138<br>(54 – 248)	< 0.001
Serum transferrin [mg/dl]	79	315.6<br>(± 69.7)	60	265.7<br>(± 59.2)	< 0.001
Transferrin saturation [%]	79	6<br>(4 - 11)	60	16<br>(9 – 23)	< 0.001
Calcium [mmol/L]	64	2.21<br>(2.08 – 2.3)	57	2.22<br>(2.13 – 2.3)	0.428
Phosphate [mmol/L]	81	0.97<br>(0.91 – 1.15)	81	0.93<br>(0.7 – 1.15)	0.005
Parathyroid hormone [ng/L]	34	36.6<br>(27.1 – 56.1)	29	37.2<br>(25.8 – 57.8)	0.923
iFGF-23 [pg/ml]	34	57.6<br>(43.1 – 77.9)	29	61.9<br>(43.4 – 84.7)	0.792
cFGF-23 [pmol/L]	34	2.5<br>(0.8 – 9.5)	29	1.3<br>(0.6 – 3.6)	< 0.001
25 (OH) vitamin D3 [nmol/L]	34	36.3<br>(22.2 – 67.6)	29	37.3<br>(17.8 – 74.5)	0.657
1,25 (OH) vitamin D3 [pg/ml]	34	37.3<br>(19.2 – 68.6)	29	36.4<br>(18 – 55)	0.889
Alkaline phosphatase [U/L]	78	69<br>(57 – 96.3)	78	78<br>(59 – 104)	0.004

Table B:

	n	No hypophosphatemia after i.v. iron		Hypophosphatemia after i.v. iron	p
n (females)		55 (30)		25 (10)	0.235
age	55	55.2 (± 20.6)	26	45.8 (± 15.1)	0.041
Crohn's disease<br>Ulcerative colitis<br>Occult blood loss<br>Other	55	14.5 % (8)<br>23.6 % (13)<br>29.1 % (16)<br>32.8 % (18)	26	34.6 % (9)<br>23.1 % (6)<br>15.4 % (4)<br>26.9 % (7)	0.177
Ferric Carboxymaltose<br>Iron Isomaltoside 1000	55	54.5% (30)<br>45.5% (25)	26	96.2% (25)<br>3.8% (1)	< 0.001
Dose<br>0.5 g<br>1 g<br>>1 g	55	34.5 % (19)<br>61.8 % (34)<br>3.6 % (2)	26	11.5 % (3)<br>84.6 % (22)<br>3.8 % (1)	0.092
Serum ferritin [µg/L]	54	12 (8 – 30)	25	17 (9.5 – 29)	0.623
Hemoglobin [g/L]	55	92 (82 – 108)	26	107.5 (98.5 – 121.8)	0.004
Phosphate (mmol/L)	55	1.07 (± 0.19)	26	0.90 (± 0.16)	< 0.001
Phosphate (mmol/L) w/o a priori hypophosphatemia	53	1.05 (0.95 – 1.2)	19	0.95 (0.91 – 1.05)	0.005
cFGF-23 [pmol/L]	21	3.3 (0.7 – 24.5)	13	1.89 (1 – 5.9)	0.357
iFGF-23 [pg/ml]	21	62.2 (± 19.3)	13	61.4 (± 28.8)	0.926
25 (OH) vitamin D3 [nmol/L]	21	42 (± 25)	13	51 (± 34.4)	0.389
1,25 (OH) vitamin D3 [pg/ml]	21	34.2 (19 – 46.8)	13	56.6 (14.5 – 83.6)	0.228
Parathyroid hormone [ng/L]	21	34 (27 – 52.7)	13	43.3 (34.8 – 56,7)	0.208
Calcium (mmol/L)	43	2.2 (± 0.1)	21	2.2 (± 0.1)	0.468
Alkaline phosphatase [U/L]	54	71 (54.8 - 102)	25	66 (60 – 87.5)	0.373

Table C:

	n	No hypophosphatemia after i.v. iron	n	Hypophosphatemia after i.v. iron	p
Delta serum iron (µmol/L)	39	4.9 (0.3 – 7.6)	21	3.4 (1.2 – 12.9)	0.676
Delta serum ferritin (µg/L)	39	80 (9 – 145)	21	160 (97 – 286.5)	0.007
Delta serum transferrin (mg/dl)	38	-61.5 (-111 – -12.5)	21	-55 (-88 – -21.5)	0.728
Delta transferrin saturation (%)	38	6.5 (-0.3 – 15.3)	21	9 (2.5 – 23)	0.375
Delta hemoglobin (g/L)	54	19.5 (7.7 – 29.3)	26	4 (-4.3 – 20.8)	0.009
Delta phosphate (mmol/L)	55	0.02 (± 0.21)	26	-0.37 (± 0.25)	<0.001
Delta Phosphate (mmol/L) w/o a priori hypophosphatemia	53	0.01 (± 0.2)	19	-0.45 (± 0.22)	<0.001
Delta cFGF-23 [pmol/L]	15	-2.6 (-20.8 – 0)	13	-0.8 (-5 – -0.1)	0.300
Delta iFGF-23 [pg/ml]	19	-9.7 (-19.5 – 7.3)	8	34.4 (-6.6 – 76.1)	0.049
Delta 25 (OH) vitamin D3 [nmol/L]	15	4.1 (-8.6 – 22.4)	13	-11 (-41.5 – 0.5)	0.065
Delta 1.25 (OH) vitamin D3 [pg/ml]	15	0 (-13.2 – 13.9)	13	-7.4 (-53.3 – 8)	0.269
Delta Parathyroid hormone [ng/L]	15	-2.9 (-11.1 – 7.6)	13	-16.7 (-36.9 – 0.5)	0.189
Delta Calcium (mmol/L)	38	-0.02 (-0.12 – 0.01)	16	0.03 (-0.09 – 0.2)	0.144
Delta alkaline phosphatase [U/L]	53	4 (-2 – 16.5)	25	1 (-5.5 – 11.5)	0.193

Table D:

	n	FCM	n	IIM	p
Serum iron [µmol/L]	45	10.6<br>(6.8 - 17)	16	7.4<br>(4.8 – 13.2)	0.123
Serum ferritin [µg/L]	45	164<br>(60 – 255)	16	78.5<br>(37 – 159.8)	0.121
Serum transferrin [mg/dl]	45	261.9<br>(± 62.2)	15	276.9<br>(± 49.3)	0.400
Transferrin saturation [%]	45	17<br>(10 – 24.5)	15	12<br>(5 – 19)	0.134
Hemoglobin [g/L]	54	118.8<br>(± 17.8)	25	108.8<br>(± 18.5)	0.024
Phosphate (mmol/L)	55	0.82<br>(± 0.323)	26	1.11<br>(± 0.26)	<0.001
Phosphate (mmol/L) w/o a priori hypophosphatemia	47	0.85<br>(± 0.33)	25	1.11<br>(± 0.27)	0.001
cFGF-23 [pmol/L]	24	1.29<br>(0.65 – 3.3)	5	2.4<br>(0.53 – 13.5)	0.773
iFGF-23 [pg/ml]	24	59.1<br>(39.5 – 87.1)	5	62.6<br>(48.7 – 118.1)	0.564
25 (OH) vitamin D3 [nmol/L]	24	32.7<br>(17.6 – 71.3)	5	53.7<br>(22 – 86.3)	0.564
1,25 (OH) vitamin D3 [pg/ml]	24	37.2<br>(21.6 – 63.3)	5	19.4<br>(12 – 44)	0.272
Parathyroid hormone [ng/L]	24	37.6<br>(27.6 – 59)	5	37.2<br>(15 – 73.1)	0.644
Calcium (mmol/L)	37	2.18<br>(± 0.2)	20	2.22<br>(± 0.09)	0.323
Alkaline phosphatase [U/L]	55	74<br>(55 – 100)	24	80.5<br>(70.5 – 112.3)	0.158
Hypophosphatemia:<br>normal (≥0.8 mmol/L)<br>moderate (<0.8 mmol/L)<br>severe (<0.6 mmol/L)<br>life-threatening (<0.3 mmol/L)	55	 54.5% (30)<br>12.7% (7)<br>29.1% (16)<br>3.6% (2)	26	 96.2% (25)<br>3.8 % (1)<br>0 % (0)<br>0 % (0)	0.002

Table E:

	n	FCM	n	IIM	p
Delta hemoglobin (g/L)	55	16 (2-26)	25	14 (7 - 25)	0.807
Delta serum iron (µmol/L)	44	4.6 (0.6 – 10.8)	16	2.6 (-0,7 – 9.3)	0.707
Delta serum ferritin (µg/L)	44	136 (29.3 – 235.7)	16	60.5 (10.8 - 128)	0.082
Delta serum transferrin (mg/dl)	44	-56 (-90.5 – -16.3)	15	-81 (-126 – -15)	0.370
Delta transferrin saturation (%)	44	8.5 (2 – 19,5)	15	6 (3 – 16)	0.502
Delta phosphate (mmol/L)	55	- 0.17 (± 0.31)	26	0.26 (± 0.21)	0.005
Delta alkaline phosphatase [U/L]	55	3 (-5 – 9)	23	10 (0 – 32)	0.038
